# Student perspectives on health dialogues: how do they benefit?

**DOI:** 10.1080/17482631.2021.1876614

**Published:** 2021-01-21

**Authors:** Malin Rising Holmström, Lena Boström

**Affiliations:** a Department of Nursing, Mid Sweden University, Sundsvall, Sweden; b Department of Education, Mid Sweden University, Sundsvall, Sweden

**Keywords:** Child’s perspective, qualitative content analysis, health dialogue, nursing, school environment, students’ health and learning

## Abstract

The vast majority of students in Sweden are healthy and live good lives, but rising health problems and declining academic performance pose risks to the country’s student population. School health services (SHS) in Sweden have a long tradition of hosting health dialogues (HD) with students to support their health and its repercussions on their learning.

**Purpose**: To describe experiences from 6- to 16-year-old students participating in the health dialogue in school.

**Method**: Descriptive qualitative design. Data were collected from 734 open-ended responses on a questionnaire distributed among students ranging in age from 6 to 16. The data were subjected to qualitative content analysis.

**Findings**: The analysis identified five categories: *Important to identify health and health problems, School nurse, a key profession, School environment important for both health and learning, Importance of health screening* and *Important to have a healthy lifestyle*. The findings revealed that students aged 6 to 16 years old consider health and health problems, school environment, health screening and healthy lifestyle to be important areas for health and learning School nurses were identified as key persons in HD and for student’s experience of health and learning.

**Conclusion**: HD is an opportunity for students to participate and influence decisions concerning their health and education, to actively engage in their own health and learning in areas that affect them for example, the school environment.

## Introduction

### Health and learning

The association between student’s health and learning has been highlighted by research and authorities (Skolverket, 2019a). Health behaviours learnt during childhood tend to persist throughout life. Childhood is an important period not only for physical, mental, social development, establishing good health behaviours and a healthy lifestyle, but also an important period for learning (Faresjo & Rahmqvist, 2010; Hjörne & Säljö, 2014; Petersen, 2008; Specialpedagogiska skolmyndigheten, 2018).

In Sweden, the vast majority of students are healthy and live good lives (Folkhälsomyndigheten, 2019). Schools should promote students’ physical, mental and social health as well as their learning (SFS, 2010:800). However, research to date has revealed significant correlations between promoting students’ health and improving their learning (Awartani et al., 2008; Coe et al., 2006). In the past decade, education in Sweden, supported by a new grading system, has demonstrated not only reduced academic performance compared to other countries (Skolverket, 2019a, 2019b) but also a falling proportion of secondary school graduates, namely from 90% to 83% (Skolverket, 2019c). Moreover, upper secondary school has reported low throughput and a high proportion of students with gaps in coursework and a lack of motivation (Skolverket, 2019d). In fact, 53% of students at the upper secondary level reported feeling largely unmotivated (Sveriges elevkårer & Lärarnas riksförbund, 2015).

In response to those trends, the most important means to increase students’ motivation may be promoting various forms of teacher–student interaction and ensuring access to student health services (Sveriges elevkårer & Lärarnas riksförbund, 2015; SFS, 2010:800). Researchers and authorities alike have highlighted the importance of the interaction and interconnection between health and learning (Faresjo & Rahmqvist, 2010; Hjörne & Säljö, 2014; Specialpedagogiska skolmyndigheten, 2018), particularly its bidirectionality for both children and adolescents (Skolverket, 2019a). Considering those relationships, Sweden may take advantage of its long tradition of health screening, overseen by School Health Services, (SHS) and performed by school nurses at each school. In doing so, the goals of SHS are to identify health problems, take measures to prevent poor health and promote health and learning by not only performing health screening but also hosting health visits with health dialogues (HD) between students and school nurses (SFS, 2010:800; Socialstyrelsen, 2016).

### Health dialogues (HDs) in Sweden

Health dialogues as part of a concept with health visits have been used for the past two decades in Sweden’s schools, and HDs represent an approach to promoting health among students. The HD used in this study is a concept used and developed in a bottom-up perspective with and by school nurses and SHS in the north of Sweden (Rising-Holmström, 2013). There are other similar approaches with health visits used in Sweden developed in a top-down perspective by researchers (Golsäter, 2012). The objectives of the HD in this study are threefold: to increase the student’s awareness of how a healthy lifestyle is associated with health and learning, to promote health and wellbeing as well as to identify early signs of health problems (Rising-Holmström, 2013; Socialstyrelsen, 2016). Provided individually to students, an HD consists of three parts: 1. health screening with school nurse (checking of e.g., height, weight, vision and hearing) 2. responding to a questionnaire—the Health Dialogue Questionnaire (HDQ), and 3. dialogue with a school nurse that refers to the responses on the HDQ and the health screening. The HDQ is age-appropriate related to number and content in questions. The questions are related to physical, mental and social health according to development and growth from 6 to 16 years of age. To comprehend the full context of HDs, a brief explanation of how they accommodate the child perspective, the individual child’s perspective and children’s rights is provided in the following sub-sections.

### The UN convention on the Rights of the Child

As children—that is, individuals less than 18 years old—students have the right to engage in health activities that affect them, including HDs. That corresponds with the four major principles of the United Nations Convention on the Rights of the Child (CRC) (i.e., Articles 2, 3, 6 and 12), which guide the interpretation of the CRC’s other articles (UNICEF Sverige, 2009). Parts of those principles also appear in Chapter 1, Section 10, of Sweden’s Education Act (SFS, 2010), which states that in all educational and other activities that concern children, the best interests of the child have to be prioritized. The CRC also maintains that each child should be afforded the opportunity to freely express his or her views on all matters that concern him or her and that the child’s opinions should be given importance in light of his or her age and maturity level (UNICEF Sverige, 2009).

### The child perspective, the child’s perspective and the child’s rights perspective

According to Article 3 of the CRC, students’ health, as well as other activities concerning and including children and students, need to be understood from the viewpoint of children, which should highlight each child’s rights and children’s best interests from three perspectives: the child perspective, the child’s perspective and the child’s rights perspective. First, the *child perspective* means that adults who are as familiar as possible with a child’s situation need to always bear the child’s best interests in mind. By contrast, taking the *child’s perspective* means listening to and respecting the prerogatives of the child based on his or her age and maturity level and affording the child the opportunity to share his or her experiences, views and suggestions to making decisions that affect him or her. Last, the *child’s rights perspective* refers to the child’s legal status and is not a personal interpretation of what is best for the child. From that perspective, it is important that regulations at school and in social services and healthcare services are well coordinated (Golsäter, 2012; Söderbäck et al., 2011; Sommer et al., 2010; UNICEF Sverige, 2009). Students should receive opportunities to influence decisions concerning their health and education and are continually encouraged to actively engage in work concerning issues of health and are especially informed about such issues that affect them (SFS, 2010; Socialstyrelsen, 2016). Our study respected those imperatives by seeking to identify possibilities to better enable students to participate and influence HDs as an activity related to their health and learning.

### Previous research

In Sweden, the focus of SHS and the role of health visits and HDs therein have been strengthened since the implementation of the Swedish Education Act in 2010, especially by prioritizing preventative approaches that underscore learning about health and cultivating school environments that promote good health and learning (SFS, 2010:800). In parallel to the longstanding use of HDs and health visits in Sweden by school nurses, research has sustained focus on how school nurses experience the dialogues and meetings with students that they entail (Arnesdotter et al., 2008; Holmström et al., 2015, 2013b; Laholt et al., 2018; Summach, 2011), and on how students experience health visits with school nurses (Golsäter, 2012) and dialogues about mental health (Johansson & Ehnfors, 2006). Adding to that scholarship, Borup has conducted several studies on health visits in Denmark, including school nurses’ assessments of successful HDs (Borup & Holstein, 2007), how students victimized by bullying benefit from HDs (Borup, 2002) and students’ reflections on and reactions to HDs (Borup, 2007).

The health dialogue concept and questionnaire described in our study have been systematically used for more than 20 years and has been validated in several studies (Holmström et al., 2013b; Kristiansen et al., 2016; Olofsson et al., 2015). However, the health dialogue concept and questionnaire has not been validated with the input of key persons, the 6- to 16-year-old student, even though the Swedish National Agency for Education has emphasized the need to amplify the voices and stories of children and young people and their own understanding of health and learning, both in everyday work within the framework of teaching and in the work around children and young people in need of support (Skolverket, 2019a). Nevertheless, it remains unclear how students experience the content, dialogue and questionnaire of the HDs. Thus, we designed our study to promote students’ engagement in HDs, with the express aim of gaining insights into students’ unique and common experiences of participating in HD. To our knowledge, no studies have focused exclusively on the experiences of students participating in these HDs, particularly regarding context, content, dialogue and questionnaire (HDQ). Thus, the aim of this study was to describe experiences from 6–16-year- old students participating in the health dialogue in school.

## Methods

### Design

A descriptive qualitative design was used for the present study which is part of a larger research project investigating the experiences of 6- to 16-year-old students participating in health dialogues in the north of Sweden using the HDQ. For this research project an instrument to evaluate students’ experiences with HDs, an evaluation questionnaire (EQ) with 20 age-appropriated questions based on the content in the HDQ, with space for open-ended responses, was developed by a group of school nurses and with support from public health professionals in September 2016. After each question on the EQ, an empty space was provided for open-ended responses, and to obtain data as rich, detailed and complete as possible, students were encouraged to elaborate upon their previous answers or comment in their own words about their experiences with HDs A reference group of eight students aged 6- to 16 years and six parents participated in a pilot test of the EQ; the findings recommended minor linguistic corrections, and this was done accordingly. In this study, only the open-ended responses that students answered in their own words, were included which we subsequently subjected into manifest qualitative content analysis conducted and involving close scrutiny of the text and text only (Polit & Beck, 2016).

### Data collection

From September 2016 to June 2019, we collected data from responses to the open-ended questions in the EQ that students answered in their own words. One author transcribed those responses verbatim to a single text unit consisting of 38 A4-sized pages of text (i.e., in 12-point Times New Roman) in Microsoft Word, for an even distribution of comments from all participating age groups (i.e., 6-, 10-, 13- and 16-year-olds). Responses spanned two lines of text on average but varied from a few words to detailed, multi-paragraph-length answers with analyses and personal descriptions. Ultimately, 734 responses were analysed, with a gender distribution of 56% from boys and 43% from girls.

### Participants and procedures

The participating students were recruited from a municipality in northern Sweden, and all students with experience engaging in HDs at school were eligible to participate. A letter containing information about the study and explaining consent to participate, as well as the EQ and a response envelope, was distributed by the school nurses to each student in kindergarten (6y), Grade 4 (10y), Grade 7 (13y) and Grade 10 (16y), and to his or her guardian(s) when the student participated in the ordinary HD. Participation was voluntary, and students who returned completed questionnaires were interpreted as having provided their consent to participate (Polit & Beck, 2016).

### Data analysis

The data were analysed using manifest qualitative content analysis with an inductive approach according to Graneheim and Lundman (2004). The analysis started with several readings of the data to gain a sense of the content. In the next step, meaning units were identified guided by the aim of the study. The meaning units were condensed, coded and sorted into sub-categories based on similarities and differences in content. The sub-categories were compared, and categories were identified, i.e., threads of meaning that appear in category after category cf (Baxter, 1994). The transcript was then reread to refine and verify the categories (Graneheim & Lundman, 2004) (Table I). Due to ethical considerations to guarantee students’ confidentiality, the findings herein are presented for all students and not presented according to age or gender. It should be mentioned that the gender and age distributions of the open-ended responses were relatively equal. The older students’ responses were generally longer and richer in content than the younger students’ responses.

**Table I.** Examples of the analysis process

Meaning units	Condensed meaning unit	Codes	Subcategories	Categories
*“It’s important to pay attention to poor mental health”*	Poor mental health is important	Poor mental health	Important with mental health problems	Important to identify health and health problems
*‘The school nurse asks how I feel, especially mentally. That´s important`*	Important that school nurses ask about student´s health	School nurse and health	School nurse important for student´s health	School nurse, a key profession
*“I need peace and quiet in the classroom to be able to concentrate and understand”*	Peace and quite important for students´ concentration and learning	Classroom environment	Classroom environment, health and learning	School environment important for health and learning
*It is not important to weigh me because I know my weight and its nobody’s business’*	Not important for students to be weighed	Weight control	Weight controls out of date	Importance of health screening
*“We talked about how much unhealthy stuff, such as sweets, you eat. I’ve eaten fewer sweets since the health dialogue”*	Student talked about unhealthy food in HD and reduced eating sweets	Eating healthy food	Healthy lifestyle	Important with a healthy lifestyle

### Trustworthiness

To achieve trustworthiness (credibility, dependability, confirmability and transferability), we formed a purposeful sample based on selection criteria thought to enrich the variation of the phenomenon being studied. This was an expression for supporting credibility of the study. One strength of the study was the rich material of 743 open-ended responses originated from students age 6–16 years old. Due to ethical considerations to guarantee students’ confidentiality, the findings herein were presented for all students and not presented according to age or gender and this on the other hand could be considered a limitation of the study. It should be mentioned that the gender and age distributions of the open-ended responses were relatively equal. The older students’ responses were generally longer and richer in content than the younger students’ responses. Furthermore, we have described the different steps taken during analysis and the research process, as well as presented verbatim quotations in the findings, all of which contribute to the trustworthiness and confirmability of the findings and enable readers to assess their validity. Dependability was supported in the way the authors worked closely throughout the analysis and agreed on the findings. Transferability’ includes the extent to which the findings of the study is transferable to other groups. Transferability’ includes the extent to which the findings of the study is transferable to other groups. However, the potential for transferability can be increased by including as many varied cases of the same phenomenon as possible in the study and in this study, there were 743 open-ended responses. However, the findings from this study cannot be generalized, but might cautiously be transferred to others in similar situations or context (Graneheim & Lundman, 2004; Polit & Beck, 2016) (Table I).

### Ethics

The study was approved by the head of education in the municipality where the study was conducted as well as by the Ethical Review Agency in Sweden (2008–122 M, 2013/91-31) and was conducted according to the ethical principles recommended by the Research Council.

## Findings

The aim of this study was to describe experiences from 6–16-year-old students participating in health dialogues in school. In the following, we present the findings from the analysis of the 734 open-ended responses from the evaluation questionnaire (EQ).

Five categories were identified during the analysis: *Important to identify health and health problems, School nurse, a key profession, School environment important for both health and learning, Importance of health screening* and *Important to have a healthy lifestyle.*

The categories were described in the text with quotations from the following paragraphs.

### Important to identify health and health problems

This was the largest category and contained both brief, fragmentary answers and longer, explanatory ones, including, *“It’s important to pay attention to poor mental health”* and *“HDs help me to overcome my social anxiety and my panic anxiety”*. In the category, several answers emphasized the importance of mental health and students all ages experienced that it was important to talk about mental health, even though they did not have mental health problems themselves. Students expressed feeling reassured knowing that if they would have health problems in the future they would get help in the HD *“HDs help and support students health”*. There were also experiences that emphasized the importance of confirming good health as well as students own thoughts about their health. *It was reassuring to know that I’m healthy*’. Students also expressed that HD was an opportunity for them to freely express his or her views on their own health that concern him or her and that the student’s opinions should be given importance “*In HDs, I talk about my health, how I feel is important to me not just my parents”*.

### School nurse, a key profession

This category contained the most detailed descriptions of all, not only about the school nurse’s role and function but also the opportunity to have such interactions with a health professional. The nurse’s function was addressed in several responses, including “*If you don’t feel well, then there’s help”*. The encounter with the nurse was very important for the outcome of the HD and there were some negative examples when the student did not experience that the nurse listen to them, for example, “*The school nurse did not listen to me when I talked about my problems, she just went on with her own agenda”*. But above all, the content in the category contained descriptions of the value of having an individual health dialogue with the nurse. In those responses, students expressed the importance of having someone listen to them and of having the opportunity to *“talk about things”*, to tell the nurse what was on their minds, to obtain answers to different questions, to receive personalized attention and “*To be able to trust someone and get an honest response”*. The responses also included comments on the school nurse’s sense of confidentiality as the HDs’ basis for trust. “*The school nurse asked if I felt physically and mentally well and she really listened to me and my opinions”.*

### School environment important for both health and learning

In this category the content addressed areas at various levels at school, ranging from the individual and the classroom to the school itself and the overall learning environment. Facilities mentioned included restrooms, locker rooms and halls, while trends mentioned included comradery, bullying and thriving in terms of wellbeing at school and in classrooms, as well as overall trends and attitudes towards the school and the learning environment—for example, *“My friends are the best in school*” and, *“It’s important what you think and feel about the school*”. The category also comprised content related to the safety and security in the school environment and especially no tolerance of bullying. It was important that the school environment was fair and clearly did not tolerate bullying not only when it came to bullying of individual *students but bullying in general. “No one feels good being in an environment that allows bullying*”. To have peace and quiet in the classrooms was important for concentration and learning. Students described getting headaches if there were to much noise in the classroom and also getting stressed because they got behind in the schoolwork and had to do more work at home. *“I need peace and quiet in the classroom to be able to concentrate and understand”.*

### Importance of health screening

In this category the responses were fairly brief and typically addressed the screening part such as vision, hearing, weight and height controls along with general health checks. Students experienced that health checks and screening contribute to feeling of security, confirmation and relief if the controls were without remark. The importance of health screening was sometimes questioned by students, especially the weight control was commented on by the older students as *“It is not important to weigh me because I know my weight and its nobody’s business”* and *“I simply refuse to stand on the scale. It makes me feel uncomfortable”*. Hearing and vision control was perceived as a support as this helped students for example, to get glasses and, in this way, facilitate school and everyday life. *“I got to know that I needed glasses at the HD, the glasses really help me in my schoolwork”*

### Important to have a healthy lifestyle

The response showed that health habits were experienced as important and relevant in the HD. The responses ranged from healthy living in general to more specific areas such as alcohol use, eating habits, sex habits, drug use, all sorts of different diets, habits for physical activity, sleep and questions about nutrition (what is healthy to eat and what is not and how much?). The importance of good sleep habits and a healthy lifestyle were also mentioned in many of the answers. Some of the responses indicated student’s high competence and interest in the subject, especially regarding different diets (low in carbohydrates and high in protein, no sugar, no fat, vegetarian, vegan, lactose free, gluten), and intense physical activity 6–7 days a week. The responses about alcohol, smoking and drugs showed that students had knowledge about the risks but also revealed that it sometimes was hard for students to say no when friends party. Quotations exemplifying this category were “*The most important health habit is diet. That’s because it’s most important for students” performance*’, “*I don’t eat carbs and exercise 6 days a week*”, “*I cut down on sugar after the HD*”, “*I try to be physically active every day because it helps me sleep better*” and “*I hate smoking*”, “*All my friends party. I don’t want to … but it is hard to say no*”.

## Discussion

In the following, the findings will be discussed in the light of the aim. Clearly,

students experienced various support from the HD regarding how to identify health and health problems and to live healthier lives. Many of their responses addressed eating and diet (e.g., eating more nutritious foods, consuming less sugar and sweets and eating more fruits and vegetables). HDs also seemed to furnish sound advice concerning the importance not only of physical activity but also of being physical active as well as the importance of a good learning environment in school, having friends and not being exposed for bullying. The findings indicate that HDs at schools gave students support, and opportunity to talk and participate in dialogues regarding their own health, lifestyle and academic performance.

### Important to identify health and health problems

According to the open-ended responses, it was important to identify health and health problems, especially mental health. Topics regarding mental health and poor mental health among students represent growing trends in both research and media due to increased levels of students in Sweden with mental health problems. For example, two systematic literature reviews have shown that self-reported psychosomatic disorders have increased since the 1980s, partly in high-income countries in the Western world, including Sweden (Bor et al., 2014) and in northern Europe (Potrebny et al., 2017). Studies in Sweden have confirmed that problems such as depression, anxiety and insomnia have become increasingly common among youth in Sweden in recent decades. In addition, Sweden’s National Board of Health and Welfare (i.e., Socialstyrelsen) showed that psychiatric diagnoses—above all, depression, various anxiety disorders and addiction linked to mental illness among children and young people in Sweden have increased from 2006 to 2016 (Socialstyrelsen, 2017). However, our findings do not imply that students, at least in our sample, suffer from mental health problems, only that they agreed that mental health and poor health are important topics to discuss during HDs. Therefore, we recommend taking students’ perspectives seriously and further emphasizing mental health and poor health in the HD.

### School nurse a key profession

The description of school nurse contains details about the school nurse’s role and function but also about the content of HDs and the opportunity to have such interactions with a health professional. The nurse’s function was addressed in several responses, although, above all, the content in the category contained descriptions of the value of HDs. In particular, students expressed the importance of having someone listen to them and of having the opportunity to tell the nurse what was on their minds and obtain answers to different questions. The responses also included comments on the school nurse’s sense of confidentiality as the HD’s basis for trust. Those findings correspond well with the findings of earlier research (Borup, 2002, 2007; Golsäter, 2012). However, to be able to support student in different areas, the school nurses need to be updated with a broad and deep competence. School nurses are alone when meeting the students, and earlier research has shown that school nurses experienced a burden in their work and wished for increased collaboration with other professionals (Holmström et al., 2013a). Therefore, we recommend possibilities for increased collaboration between school nurses and teachers and possibilities for school nurses to develop their skills (for example, diets and nutrition) so the school nurses can better support the students.

### School environment important for health and learning

Another area of interest for the students was the school environment, interpreted from several perspectives and encompassing the level of the student, the group and the school. In other studies, the school environment has been found to impact learning and health, especially associated with psychosomatic disorders and bullying. Sweden’s Public Health Authority has investigated possible causes for the increase in psychosomatic disorders among Swedish students, and among the probable causes were deficiencies in the functioning of the school and the school environment, which have led to impaired school performance and widespread school stress among students (Folkhälsomyndigheten, 2018; Socialstyrelsen, 2017). The school environment should promote both health and learning, as well as safety and security, for students. One way to reach those goals is to initiate solid, broad cooperation between school nurses, teachers and students regarding students´ health and learning. Participating students also emphasized the importance of having friends, close relationships and not being exposed to bullying in school. The findings show that peace and quiet in classrooms were important for concentration, learning, avoiding headaches and stress This was important for thriving, feeling comfortable and at ease in school and classrooms and also for overall academic performance and health. Furthermore, previous research on students victimized by bullying has reported particular benefits of HDs for such students (Borup & Holstein, 2007). Therefore, we recommend taking students’ perspectives seriously and seeking to fully comprehend the risks and consequences of poor learning environments in school, both for health and for learning.

### Importance of health screening

Most students experienced that health checks and screening contribute to feeling of security and confirmation of health, but some students questioned the health screening, especially weight control made the students feel uncomfortable and refuse to stand on the scale. These are very important to acknowledge though many students today are preoccupied with thoughts about not being good enough. Meeting students to talk about the body, health and weight and performing weight controls requires great sensitivity and competence (Socialstyrelsen, 2016, 2019). The findings might be interpreted that health screening is still important and contribute to student’s health, but especially weight control requires high competence and sensitivity from school nurses.

### Important with a healthy lifestyle

The students described health habits and healthy lifestyle as important areas in HDs and described a large variation in the habits discussed. It ranged from healthy lifestyles in general, such as brushing your teeth and eating vegetables, to more specific areas such as alcohol use, sex habits and birth control, intimate relationships, drug use, physical activity, sleep and nutrition. These findings show great variation and depth in health issues in the HD content, requiring new broad and updated competence and also professionalism from school nurses (SFS, 2010; Socialstyrelsen, 2016).

### Implications and directions for future research

As society in Sweden and abroad continues to change rapidly, students have become an increasingly vulnerable group. During childhood and adolescence, as attitudes and personality are formed, different perceptions of what is right and wrong, as well as healthy and harmful, can engender behavioural patterns that can pose great risks to long-term physical and mental health. Coupled with the demands of schoolwork, the development of children and adolescents can coincide with poor academic performance that can also pose serious consequences for life and future health. In response, and in light of our study’s findings, we argue for the need to further develop the collaboration between teaching staff and school health services in order to realize the full potential of the association between health and learning. Detecting early signs of poor health and/or poor academic performance in HDs, for example, may inspire preventative efforts and underscore the need for a broad approach and for mapping health habits and lifestyles among students. This present study is part of a larger research project investigating the experiences of 6- to 16-year-old students participating in health dialogues in the north of Sweden. The findings in the study additionally have not only clinical, educational and organizational implications but also implications for policy and policymaking. Because the Education Act (SFS, 2010:800) stipulates that work concerning students’ health should primarily promote health and be preventative in nature, such work should be systematic and led by the principal of each school and a student health team to support functions that have to be developed for such work. Therein, a particular challenge is developing methods that enable possibilities for interprofessional work. To that end, the collaboration and contact between student health teams and teachers need to be developed (Skolverket, 2019a). Because health and learning are so intertwined, we also believe that now is the time to review and reform policy governing schools’ work concerning students’ health in order to elucidate the importance of health in students’ academic functioning and performance. Our study has revealed that students aged 6 to 16 years old consider health and health problems, school nurse, school environment, health screening and healthy lifestyle to be important areas for health and learning. Furthermore, HD is an opportunity for students to participate and influence decisions concerning their health and education, to actively engage in their own health and learning in areas that affect them for example, the school environment.
